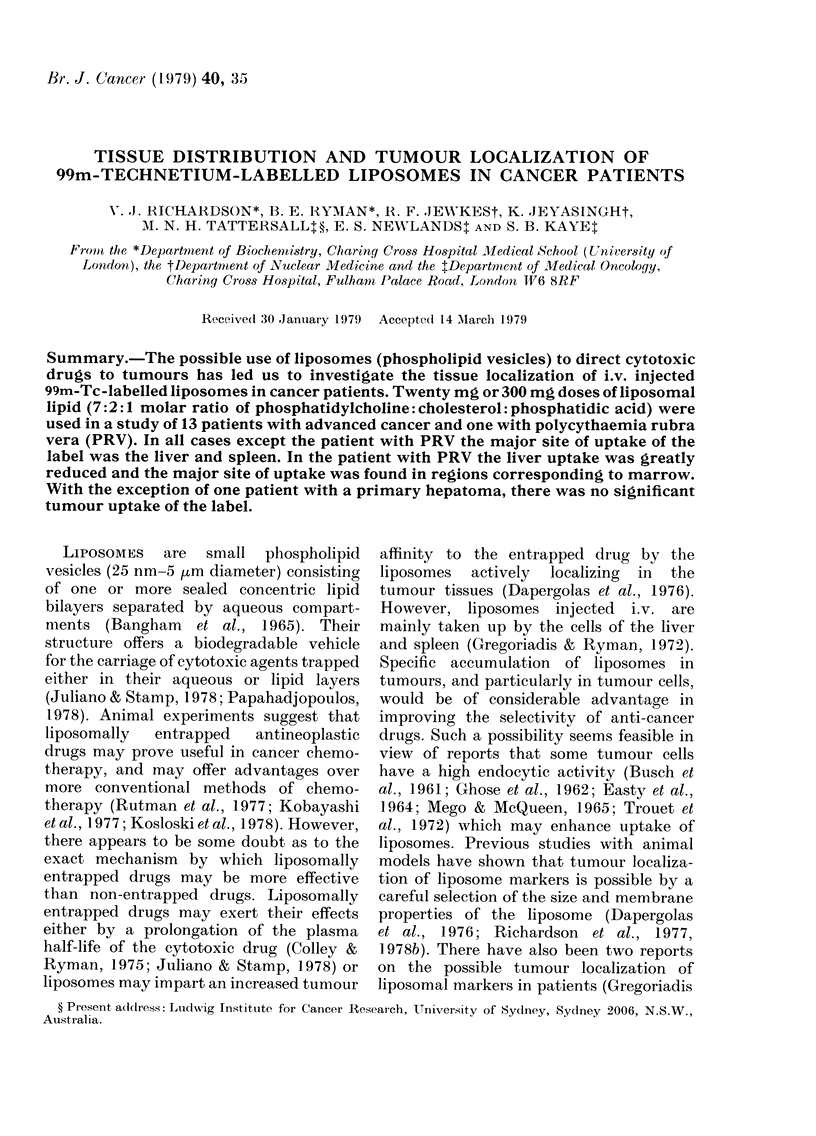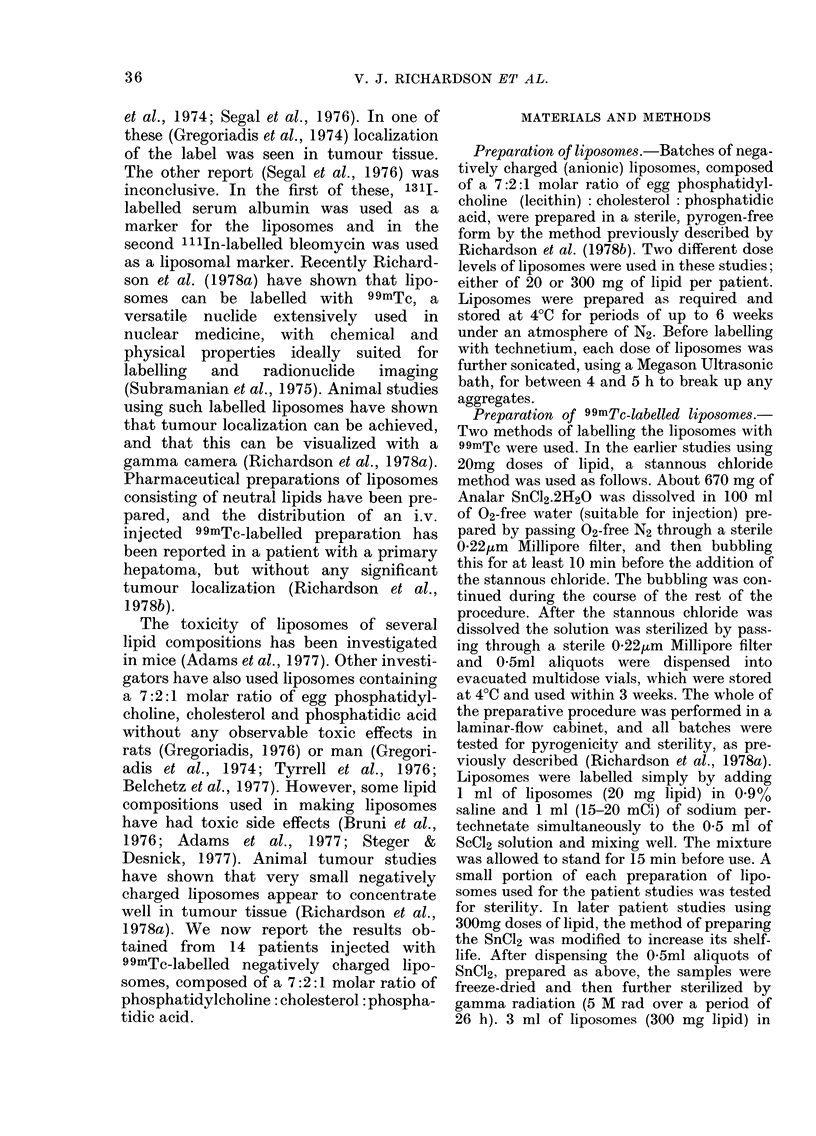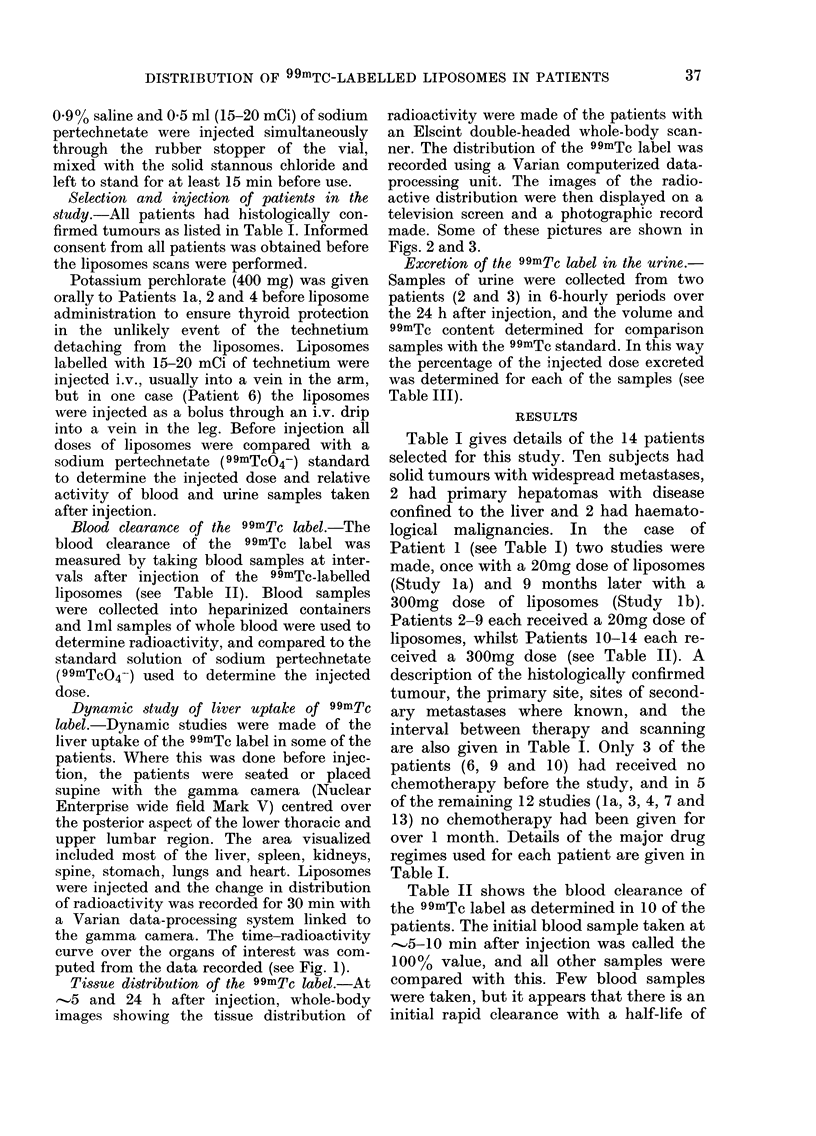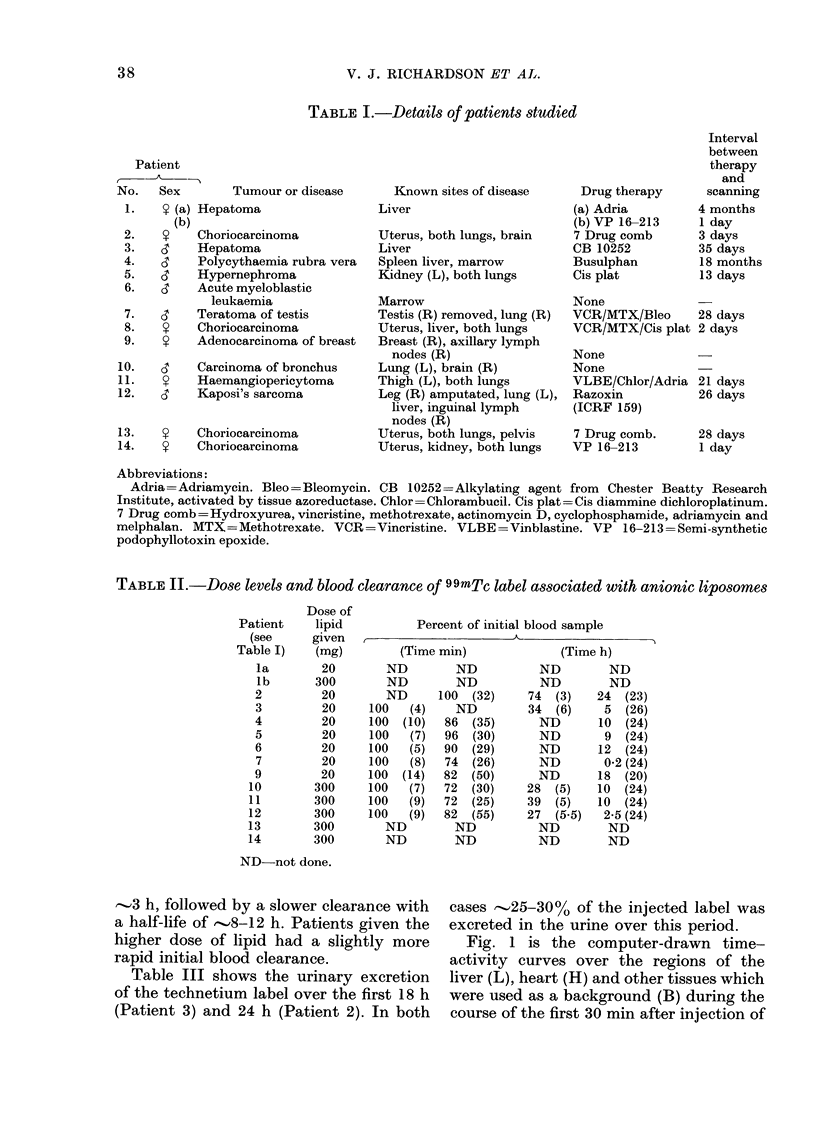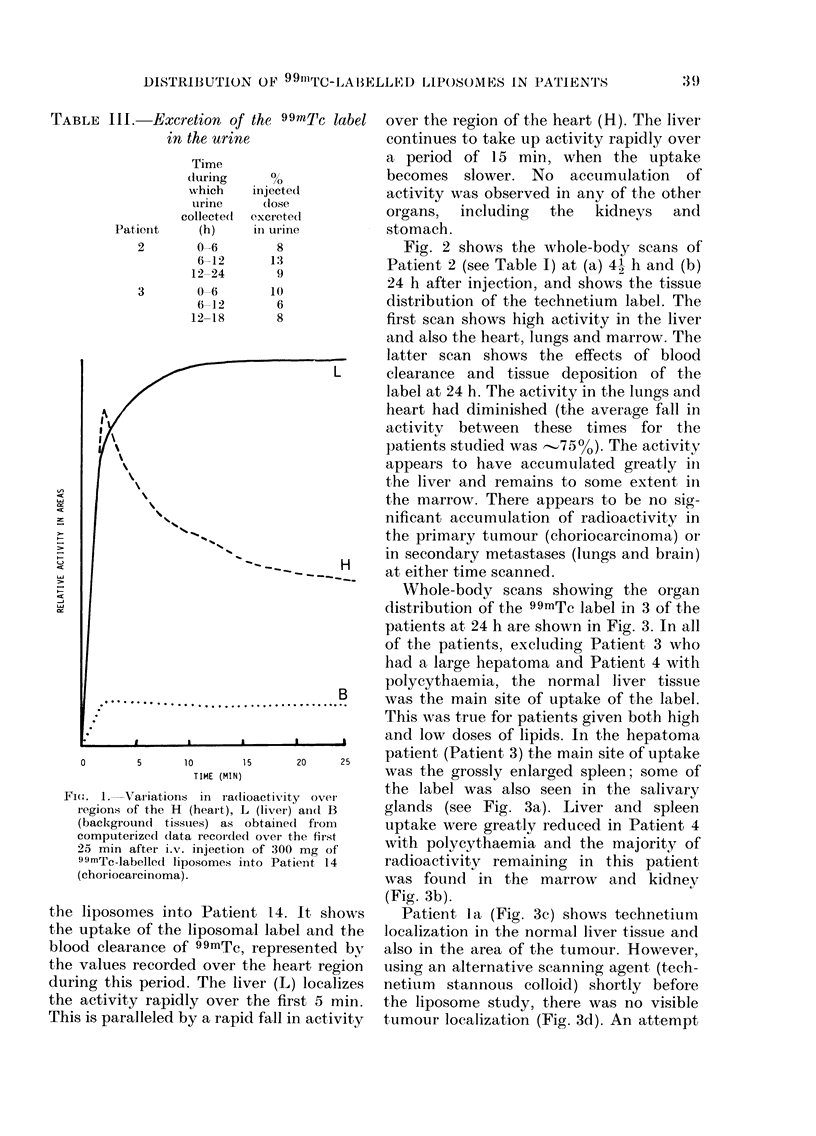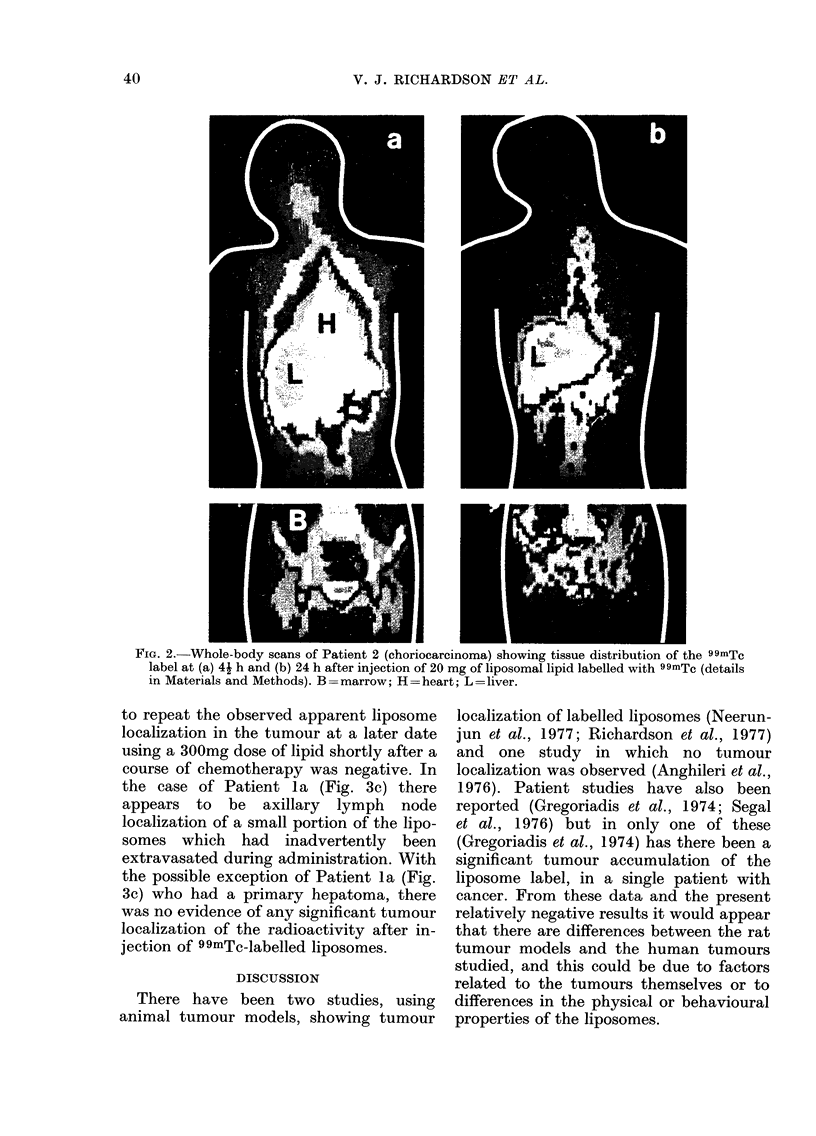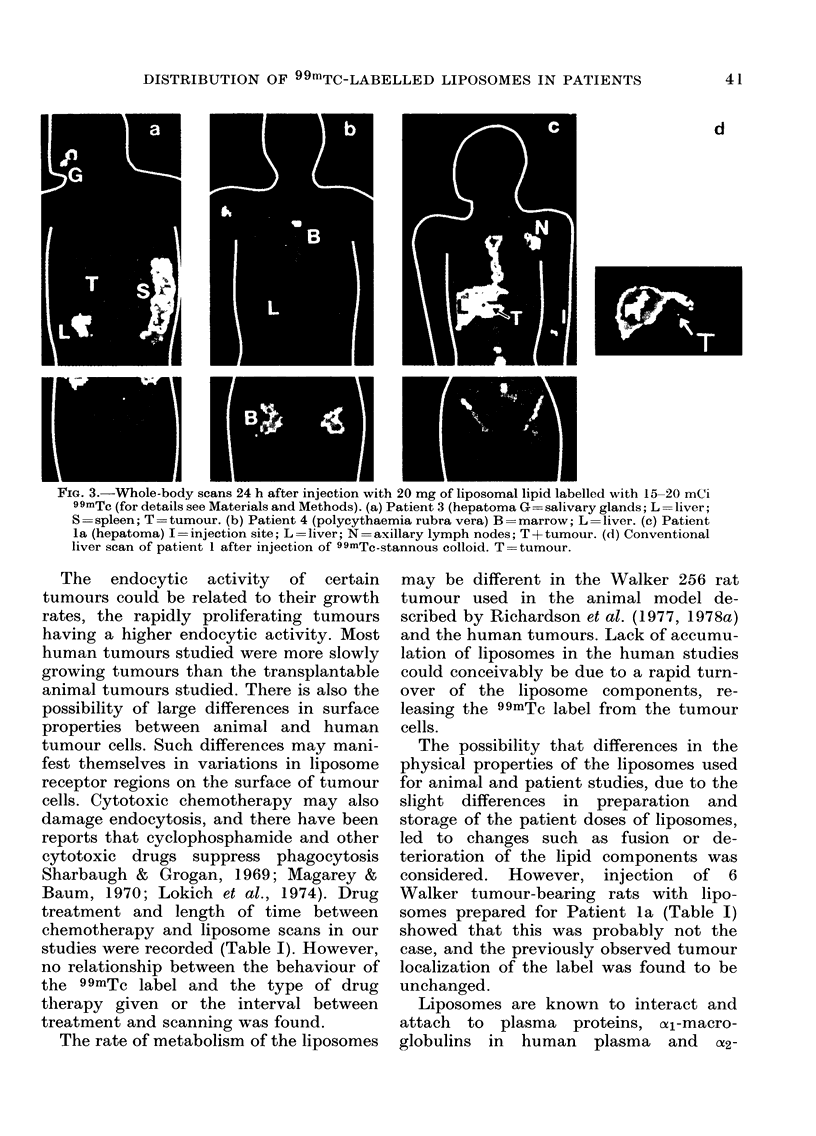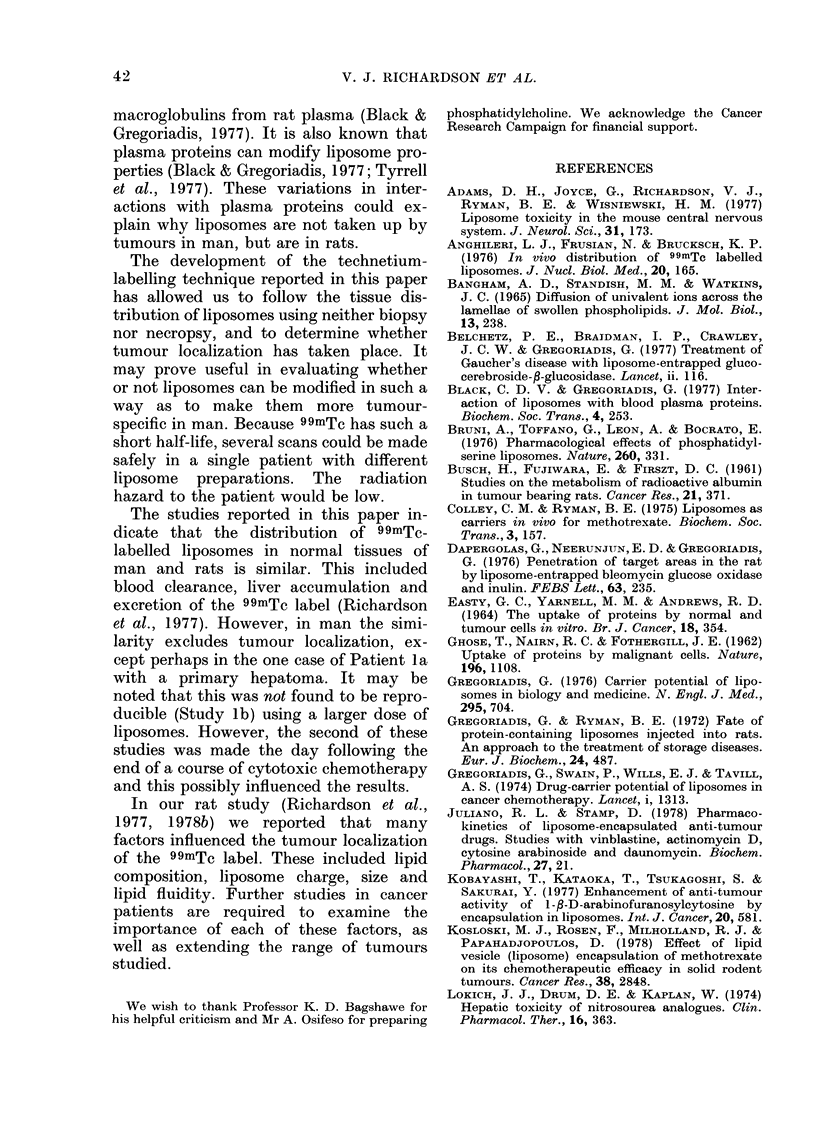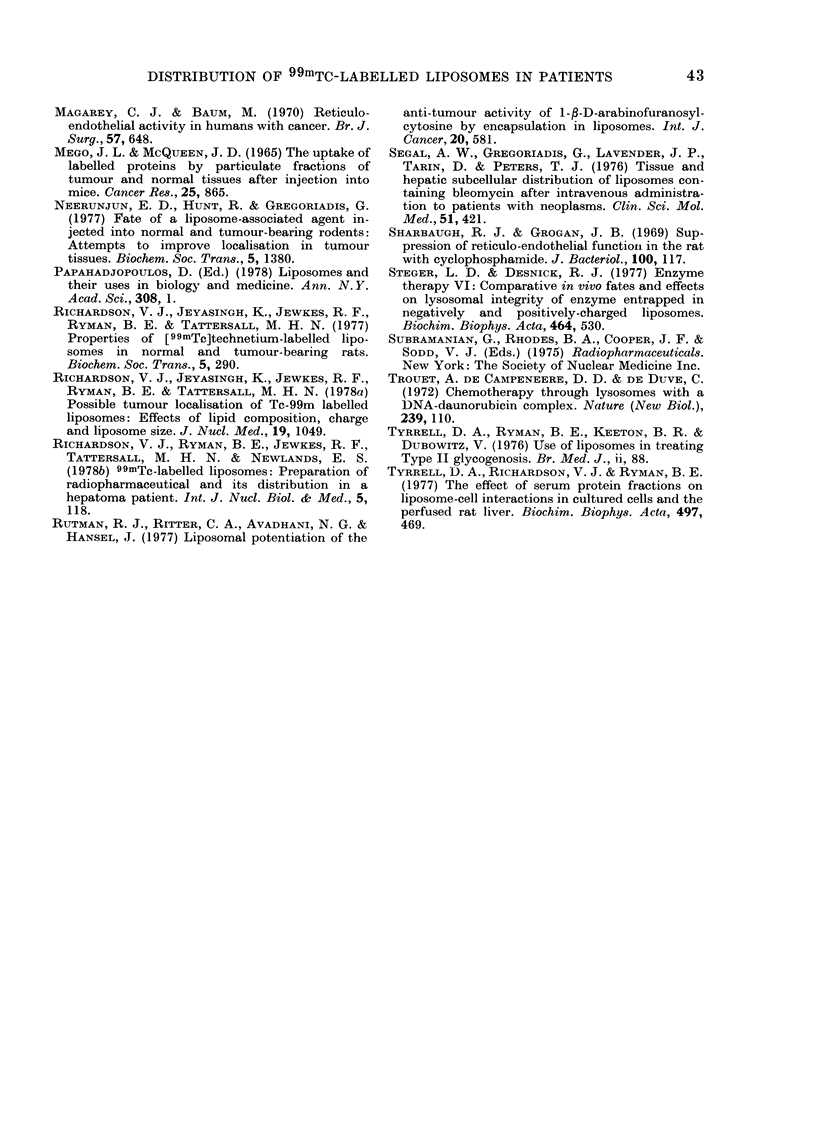# Tissue distribution and tumour localization of 99m-technetium-labelled liposomes in cancer patients.

**DOI:** 10.1038/bjc.1979.138

**Published:** 1979-07

**Authors:** V. J. Richardson, B. E. Ryman, R. F. Jewkes, K. Jeyasingh, M. N. Tattersall, E. S. Newlands, S. B. Kaye

## Abstract

**Images:**


*Br. J. Cancer* (1979) **40**, 35

### TISSUE DISTRIBUTION AND TUMOUR LOCALIZATION OF 99m-TECHNETIUM-LABELLED LIPOSOMES IN CANCER PATIENTS

V. J. RICHARDSON*, B. E. RYMAN*, R. F. JEWKES†, K. JEYASINGH†, M. N. H. TATTERSALL‡§, E. S. NEWLANDS‡ AND S. B. KAYE‡

*From the *Department of Biochemistry, Charing Cross Hospital Medical School (University of London), the †Department of Nuclear Medicine and the ‡Department of Medical Oncology, Charing Cross Hospital, Fulham Palace Road, London W6 8RF*

Received 30 January 1979 Accepted 14 March 1979

**Summary.—The possible use of liposomes (phospholipid vesicles) to direct cytotoxic drugs to tumours has led us to investigate the tissue localization of i.v. injected 99m-Tc-labelled liposomes in cancer patients. Twenty mg or 300 mg doses of liposomal lipid (7:2:1 molar ratio of phosphatidylcholine: cholesterol: phosphatidic acid) were used in a study of 13 patients with advanced cancer and one with polycythaemia rubra vera (PRV). In all cases except the patient with PRV the major site of uptake of the label was the liver and spleen. In the patient with PRV the liver uptake was greatly reduced and the major site of uptake was found in regions corresponding to marrow. With the exception of one patient with a primary hepatoma, there was no significant tumour uptake of the label.**

LIPOSOMES are small phospholipid vesicles (25 nm–5 $\mu$m diameter) consisting of one or more sealed concentric lipid bilayers separated by aqueous compartments (Bangham *et al.*, 1965). Their structure offers a biodegradable vehicle for the carriage of cytotoxic agents trapped either in their aqueous or lipid layers (Juliano & Stamp, 1978; Papahadjopoulos, 1978). Animal experiments suggest that liposomally entrapped antineoplastic drugs may prove useful in cancer chemotherapy, and may offer advantages over more conventional methods of chemotherapy (Rutman *et al.*, 1977; Kobayashi *et al.*, 1977; Kosloski *et al.*, 1978). However, there appears to be some doubt as to the exact mechanism by which liposomally entrapped drugs may be more effective than non-entrapped drugs. Liposomally entrapped drugs may exert their effects either by a prolongation of the plasma half-life of the cytotoxic drug (Colley & Ryman, 1975; Juliano & Stamp, 1978) or liposomes may impart an increased tumour affinity to the entrapped drug by the liposomes actively localizing in the tumour tissues (Dapergolas *et al.*, 1976). However, liposomes injected i.v. are mainly taken up by the cells of the liver and spleen (Gregoriadis & Ryman, 1972). Specific accumulation of liposomes in tumours, and particularly in tumour cells, would be of considerable advantage in improving the selectivity of anti-cancer drugs. Such a possibility seems feasible in view of reports that some tumour cells have a high endocytic activity (Busch *et al.*, 1961; Ghose *et al.*, 1962; Easty *et al.*, 1964; Mego & McQueen, 1965; Trouet *et al.*, 1972) which may enhance uptake of liposomes. Previous studies with animal models have shown that tumour localization of liposome markers is possible by a careful selection of the size and membrane properties of the liposome (Dapergolas *et al.*, 1976; Richardson *et al.*, 1977, 1978*b*). There have also been two reports on the possible tumour localization of liposomal markers in patients (Gregoriadis

§ Present address: Ludwig Institute for Cancer Research, University of Sydney, Sydney 2006, N.S.W., Australia.

*et al.*, 1974; Segal *et al.*, 1976). In one of these (Gregoriadis *et al.*, 1974) localization of the label was seen in tumour tissue. The other report (Segal *et al.*, 1976) was inconclusive. In the first of these, $^{131}$I-labelled serum albumin was used as a marker for the liposomes and in the second $^{111}$In-labelled bleomycin was used as a liposomal marker. Recently Richardson *et al.* (1978*a*) have shown that liposomes can be labelled with $^{99m}$Tc, a versatile nuclide extensively used in nuclear medicine, with chemical and physical properties ideally suited for labelling and radionuclide imaging (Subramanian *et al.*, 1975). Animal studies using such labelled liposomes have shown that tumour localization can be achieved, and that this can be visualized with a gamma camera (Richardson *et al.*, 1978*a*). Pharmaceutical preparations of liposomes consisting of neutral lipids have been prepared, and the distribution of an i.v. injected $^{99m}$Tc-labelled preparation has been reported in a patient with a primary hepatoma, but without any significant tumour localization (Richardson *et al.*, 1978*b*).

The toxicity of liposomes of several lipid compositions has been investigated in mice (Adams *et al.*, 1977). Other investigators have also used liposomes containing a 7:2:1 molar ratio of egg phosphatidylcholine, cholesterol and phosphatidic acid without any observable toxic effects in rats (Gregoriadis, 1976) or man (Gregoriadis *et al.*, 1974; Tyrrell *et al.*, 1976; Belchetz *et al.*, 1977). However, some lipid compositions used in making liposomes have had toxic side effects (Bruni *et al.*, 1976; Adams *et al.*, 1977; Steger & Desnick, 1977). Animal tumour studies have shown that very small negatively charged liposomes appear to concentrate well in tumour tissue (Richardson *et al.*, 1978*a*). We now report the results obtained from 14 patients injected with $^{99m}$Tc-labelled negatively charged liposomes, composed of a 7:2:1 molar ratio of phosphatidylcholine : cholesterol : phosphatidic acid.

### MATERIALS AND METHODS

*Preparation of liposomes.*—Batches of negatively charged (anionic) liposomes, composed of a 7:2:1 molar ratio of egg phosphatidylcholine (lecithin) : cholesterol : phosphatidic acid, were prepared in a sterile, pyrogen-free form by the method previously described by Richardson *et al.* (1978*b*). Two different dose levels of liposomes were used in these studies; either of 20 or 300 mg of lipid per patient. Liposomes were prepared as required and stored at 4°C for periods of up to 6 weeks under an atmosphere of $N_2$. Before labelling with technetium, each dose of liposomes was further sonicated, using a Megason Ultrasonic bath, for between 4 and 5 h to break up any aggregates.

*Preparation of $^{99m}$Tc-labelled liposomes.*—Two methods of labelling the liposomes with $^{99m}$Tc were used. In the earlier studies using 20mg doses of lipid, a stannous chloride method was used as follows. About 670 mg of Analar $SnCl_2.2H_2O$ was dissolved in 100 ml of $O_2$-free water (suitable for injection) prepared by passing $O_2$-free $N_2$ through a sterile 0·22$\mu$m Millipore filter, and then bubbling this for at least 10 min before the addition of the stannous chloride. The bubbling was continued during the course of the rest of the procedure. After the stannous chloride was dissolved the solution was sterilized by passing through a sterile 0·22$\mu$m Millipore filter and 0·5ml aliquots were dispensed into evacuated multidose vials, which were stored at 4°C and used within 3 weeks. The whole of the preparative procedure was performed in a laminar-flow cabinet, and all batches were tested for pyrogenicity and sterility, as previously described (Richardson *et al.*, 1978*a*). Liposomes were labelled simply by adding 1 ml of liposomes (20 mg lipid) in 0·9% saline and 1 ml (15–20 mCi) of sodium pertechnetate simultaneously to the 0·5 ml of $ScCl_2$ solution and mixing well. The mixture was allowed to stand for 15 min before use. A small portion of each preparation of liposomes used for the patient studies was tested for sterility. In later patient studies using 300mg doses of lipid, the method of preparing the $SnCl_2$ was modified to increase its shelf-life. After dispensing the 0·5ml aliquots of $SnCl_2$, prepared as above, the samples were freeze-dried and then further sterilized by gamma radiation (5 M rad over a period of 26 h). 3 ml of liposomes (300 mg lipid) in

0·9% saline and 0·5 ml (15–20 mCi) of sodium pertechnetate were injected simultaneously through the rubber stopper of the vial, mixed with the solid stannous chloride and left to stand for at least 15 min before use.

*Selection and injection of patients in the study.*—All patients had histologically confirmed tumours as listed in Table I. Informed consent from all patients was obtained before the liposomes scans were performed.

Potassium perchlorate (400 mg) was given orally to Patients 1a, 2 and 4 before liposome administration to ensure thyroid protection in the unlikely event of the technetium detaching from the liposomes. Liposomes labelled with 15–20 mCi of technetium were injected i.v., usually into a vein in the arm, but in one case (Patient 6) the liposomes were injected as a bolus through an i.v. drip into a vein in the leg. Before injection all doses of liposomes were compared with a sodium pertechnetate ($^{99m}TcO_4^-$) standard to determine the injected dose and relative activity of blood and urine samples taken after injection.

*Blood clearance of the $^{99m}$Tc label.*—The blood clearance of the $^{99m}$Tc label was measured by taking blood samples at intervals after injection of the $^{99m}$Tc-labelled liposomes (see Table II). Blood samples were collected into heparinized containers and 1ml samples of whole blood were used to determine radioactivity, and compared to the standard solution of sodium pertechnetate ($^{99m}TcO_4^-$) used to determine the injected dose.

*Dynamic study of liver uptake of $^{99m}$Tc label.*—Dynamic studies were made of the liver uptake of the $^{99m}$Tc label in some of the patients. Where this was done before injection, the patients were seated or placed supine with the gamma camera (Nuclear Enterprise wide field Mark V) centred over the posterior aspect of the lower thoracic and upper lumbar region. The area visualized included most of the liver, spleen, kidneys, spine, stomach, lungs and heart. Liposomes were injected and the change in distribution of radioactivity was recorded for 30 min with a Varian data-processing system linked to the gamma camera. The time–radioactivity curve over the organs of interest was computed from the data recorded (see Fig. 1).

*Tissue distribution of the $^{99m}$Tc label.*—At ~5 and 24 h after injection, whole-body images showing the tissue distribution of radioactivity were made of the patients with an Elscint double-headed whole-body scanner. The distribution of the $^{99m}$Tc label was recorded using a Varian computerized data-processing unit. The images of the radioactive distribution were then displayed on a television screen and a photographic record made. Some of these pictures are shown in Figs. 2 and 3.

*Excretion of the $^{99m}$Tc label in the urine.*—Samples of urine were collected from two patients (2 and 3) in 6-hourly periods over the 24 h after injection, and the volume and $^{99m}$Tc content determined for comparison samples with the $^{99m}$Tc standard. In this way the percentage of the injected dose excreted was determined for each of the samples (see Table III).

### RESULTS

Table I gives details of the 14 patients selected for this study. Ten subjects had solid tumours with widespread metastases, 2 had primary hepatomas with disease confined to the liver and 2 had haematological malignancies. In the case of Patient 1 (see Table I) two studies were made, once with a 20mg dose of liposomes (Study 1a) and 9 months later with a 300mg dose of liposomes (Study 1b). Patients 2–9 each received a 20mg dose of liposomes, whilst Patients 10–14 each received a 300mg dose (see Table II). A description of the histologically confirmed tumour, the primary site, sites of secondary metastases where known, and the interval between therapy and scanning are also given in Table I. Only 3 of the patients (6, 9 and 10) had received no chemotherapy before the study, and in 5 of the remaining 12 studies (1a, 3, 4, 7 and 13) no chemotherapy had been given for over 1 month. Details of the major drug regimes used for each patient are given in Table I.

Table II shows the blood clearance of the $^{99m}$Tc label as determined in 10 of the patients. The initial blood sample taken at ~5–10 min after injection was called the 100% value, and all other samples were compared with this. Few blood samples were taken, but it appears that there is an initial rapid clearance with a half-life of

TABLE I.—*Details of patients studied*

| Patient No. | Sex | Tumour or disease | Known sites of disease | Drug therapy | Interval between therapy and scanning |
|---|---|---|---|---|---|
| 1. | ♀ (a) (b) | Hepatoma | Liver | (a) Adria (b) VP 16–213 | 4 months 1 day |
| 2. | ♀ | Choriocarcinoma | Uterus, both lungs, brain | 7 Drug comb | 3 days |
| 3. | ♂ | Hepatoma | Liver | CB 10252 | 35 days |
| 4. | ♂ | Polycythaemia rubra vera | Spleen liver, marrow | Busulphan | 18 months |
| 5. | ♂ | Hypernephroma | Kidney (L), both lungs | Cis plat | 13 days |
| 6. | ♂ | Acute myeloblastic leukaemia | Marrow | None | — |
| 7. | ♂ | Teratoma of testis | Testis (R) removed, lung (R) | VCR/MTX/Bleo | 28 days |
| 8. | ♀ | Choriocarcinoma | Uterus, liver, both lungs | VCR/MTX/Cis plat | 2 days |
| 9. | ♀ | Adenocarcinoma of breast | Breast (R), axillary lymph nodes (R) | None | — |
| 10. | ♂ | Carcinoma of bronchus | Lung (L), brain (R) | None | — |
| 11. | ♀ | Haemangiopericytoma | Thigh (L), both lungs | VLBE/Chlor/Adria | 21 days |
| 12. | ♂ | Kaposi's sarcoma | Leg (R) amputated, lung (L), liver, inguinal lymph nodes (R) | Razoxin (ICRF 159) | 26 days |
| 13. | ♀ | Choriocarcinoma | Uterus, both lungs, pelvis | 7 Drug comb. | 28 days |
| 14. | ♀ | Choriocarcinoma | Uterus, kidney, both lungs | VP 16–213 | 1 day |

Abbreviations:
Adria = Adriamycin. Bleo = Bleomycin. CB 10252 = Alkylating agent from Chester Beatty Research Institute, activated by tissue azoreductase. Chlor = Chlorambucil. Cis plat = Cis diammine dichloroplatinum. 7 Drug comb = Hydroxyurea, vincristine, methotrexate, actinomycin D, cyclophosphamide, adriamycin and melphalan. MTX = Methotrexate. VCR = Vincristine. VLBE = Vinblastine. VP 16–213 = Semi-synthetic podophyllotoxin epoxide.

TABLE II.—*Dose levels and blood clearance of* $^{99m}Tc$ *label associated with anionic liposomes*

| Patient (see Table I) | Dose of lipid given (mg) | Percent of initial blood sample (Time min) | | (Time h) | |
|---|---|---|---|---|---|
| 1a | 20 | ND | ND | ND | ND |
| 1b | 300 | ND | ND | ND | ND |
| 2 | 20 | ND | 100 (32) | 74 (3) | 24 (23) |
| 3 | 20 | 100 (4) | ND | 34 (6) | 5 (26) |
| 4 | 20 | 100 (10) | 86 (35) | ND | 10 (24) |
| 5 | 20 | 100 (7) | 96 (30) | ND | 9 (24) |
| 6 | 20 | 100 (5) | 90 (29) | ND | 12 (24) |
| 7 | 20 | 100 (8) | 74 (26) | ND | 0·2 (24) |
| 9 | 20 | 100 (14) | 82 (50) | ND | 18 (20) |
| 10 | 300 | 100 (7) | 72 (30) | 28 (5) | 10 (24) |
| 11 | 300 | 100 (9) | 72 (25) | 39 (5) | 10 (24) |
| 12 | 300 | 100 (9) | 82 (55) | 27 (5·5) | 2·5 (24) |
| 13 | 300 | ND | ND | ND | ND |
| 14 | 300 | ND | ND | ND | ND |

ND—not done.

~3 h, followed by a slower clearance with a half-life of ~8–12 h. Patients given the higher dose of lipid had a slightly more rapid initial blood clearance.

Table III shows the urinary excretion of the technetium label over the first 18 h (Patient 3) and 24 h (Patient 2). In both cases ~25–30% of the injected label was excreted in the urine over this period.

Fig. 1 is the computer-drawn time–activity curves over the regions of the liver (L), heart (H) and other tissues which were used as a background (B) during the course of the first 30 min after injection of

TABLE III.—*Excretion of the $^{99m}Tc$ label in the urine*

| Patient | Time during which urine collected (h) | % injected dose excreted in urine |
|---|---|---|
| 2 | 0–6 | 8 |
| | 6–12 | 13 |
| | 12–24 | 9 |
| 3 | 0–6 | 10 |
| | 6–12 | 6 |
| | 12–18 | 8 |

FIG. 1.—Variations in radioactivity over regions of the H (heart), L (liver) and B (background tissues) as obtained from computerized data recorded over the first 25 min after i.v. injection of 300 mg of $^{99m}$Tc-labelled liposomes into Patient 14 (choriocarcinoma).

the liposomes into Patient 14. It shows the uptake of the liposomal label and the blood clearance of $^{99m}$Tc, represented by the values recorded over the heart region during this period. The liver (L) localizes the activity rapidly over the first 5 min. This is paralleled by a rapid fall in activity over the region of the heart (H). The liver continues to take up activity rapidly over a period of 15 min, when the uptake becomes slower. No accumulation of activity was observed in any of the other organs, including the kidneys and stomach.

Fig. 2 shows the whole-body scans of Patient 2 (see Table I) at (a) $4\frac{1}{2}$ h and (b) 24 h after injection, and shows the tissue distribution of the technetium label. The first scan shows high activity in the liver and also the heart, lungs and marrow. The latter scan shows the effects of blood clearance and tissue deposition of the label at 24 h. The activity in the lungs and heart had diminished (the average fall in activity between these times for the patients studied was $\sim$75%). The activity appears to have accumulated greatly in the liver and remains to some extent in the marrow. There appears to be no significant accumulation of radioactivity in the primary tumour (choriocarcinoma) or in secondary metastases (lungs and brain) at either time scanned.

Whole-body scans showing the organ distribution of the $^{99m}$Tc label in 3 of the patients at 24 h are shown in Fig. 3. In all of the patients, excluding Patient 3 who had a large hepatoma and Patient 4 with polycythaemia, the normal liver tissue was the main site of uptake of the label. This was true for patients given both high and low doses of lipids. In the hepatoma patient (Patient 3) the main site of uptake was the grossly enlarged spleen; some of the label was also seen in the salivary glands (see Fig. 3a). Liver and spleen uptake were greatly reduced in Patient 4 with polycythaemia and the majority of radioactivity remaining in this patient was found in the marrow and kidney (Fig. 3b).

Patient 1a (Fig. 3c) shows technetium localization in the normal liver tissue and also in the area of the tumour. However, using an alternative scanning agent (technetium stannous colloid) shortly before the liposome study, there was no visible tumour localization (Fig. 3d). An attempt

FIG. 2.—Whole-body scans of Patient 2 (choriocarcinoma) showing tissue distribution of the $^{99m}$Tc label at (a) 4½ h and (b) 24 h after injection of 20 mg of liposomal lipid labelled with $^{99m}$Tc (details in Materials and Methods). B = marrow; H = heart; L = liver.

to repeat the observed apparent liposome localization in the tumour at a later date using a 300mg dose of lipid shortly after a course of chemotherapy was negative. In the case of Patient 1a (Fig. 3c) there appears to be axillary lymph node localization of a small portion of the liposomes which had inadvertently been extravasated during administration. With the possible exception of Patient 1a (Fig. 3c) who had a primary hepatoma, there was no evidence of any significant tumour localization of the radioactivity after injection of $^{99m}$Tc-labelled liposomes.

### DISCUSSION

There have been two studies, using animal tumour models, showing tumour localization of labelled liposomes (Neerunjun *et al.*, 1977; Richardson *et al.*, 1977) and one study in which no tumour localization was observed (Anghileri *et al.*, 1976). Patient studies have also been reported (Gregoriadis *et al.*, 1974; Segal *et al.*, 1976) but in only one of these (Gregoriadis *et al.*, 1974) has there been a significant tumour accumulation of the liposome label, in a single patient with cancer. From these data and the present relatively negative results it would appear that there are differences between the rat tumour models and the human tumours studied, and this could be due to factors related to the tumours themselves or to differences in the physical or behavioural properties of the liposomes.

FIG. 3.—Whole-body scans 24 h after injection with 20 mg of liposomal lipid labelled with 15–20 mCi $^{99m}$Tc (for details see Materials and Methods). (a) Patient 3 (hepatoma G = salivary glands; L = liver; S = spleen; T = tumour. (b) Patient 4 (polycythaemia rubra vera) B = marrow; L = liver. (c) Patient 1a (hepatoma) I = injection site; L = liver; N = axillary lymph nodes; T + tumour. (d) Conventional liver scan of patient 1 after injection of $^{99m}$Tc-stannous colloid. T = tumour.

The endocytic activity of certain tumours could be related to their growth rates, the rapidly proliferating tumours having a higher endocytic activity. Most human tumours studied were more slowly growing tumours than the transplantable animal tumours studied. There is also the possibility of large differences in surface properties between animal and human tumour cells. Such differences may manifest themselves in variations in liposome receptor regions on the surface of tumour cells. Cytotoxic chemotherapy may also damage endocytosis, and there have been reports that cyclophosphamide and other cytotoxic drugs suppress phagocytosis Sharbaugh & Grogan, 1969; Magarey & Baum, 1970; Lokich *et al.*, 1974). Drug treatment and length of time between chemotherapy and liposome scans in our studies were recorded (Table I). However, no relationship between the behaviour of the $^{99m}$Tc label and the type of drug therapy given or the interval between treatment and scanning was found.

The rate of metabolism of the liposomes may be different in the Walker 256 rat tumour used in the animal model described by Richardson *et al.* (1977, 1978*a*) and the human tumours. Lack of accumulation of liposomes in the human studies could conceivably be due to a rapid turnover of the liposome components, releasing the $^{99m}$Tc label from the tumour cells.

The possibility that differences in the physical properties of the liposomes used for animal and patient studies, due to the slight differences in preparation and storage of the patient doses of liposomes, led to changes such as fusion or deterioration of the lipid components was considered. However, injection of 6 Walker tumour-bearing rats with liposomes prepared for Patient 1a (Table I) showed that this was probably not the case, and the previously observed tumour localization of the label was found to be unchanged.

Liposomes are known to interact and attach to plasma proteins, $\alpha_1$-macroglobulins in human plasma and $\alpha_2$-

macroglobulins from rat plasma (Black & Gregoriadis, 1977). It is also known that plasma proteins can modify liposome properties (Black & Gregoriadis, 1977; Tyrrell *et al.*, 1977). These variations in interactions with plasma proteins could explain why liposomes are not taken up by tumours in man, but are in rats.

The development of the technetium-labelling technique reported in this paper has allowed us to follow the tissue distribution of liposomes using neither biopsy nor necropsy, and to determine whether tumour localization has taken place. It may prove useful in evaluating whether or not liposomes can be modified in such a way as to make them more tumour-specific in man. Because $^{99m}$Tc has such a short half-life, several scans could be made safely in a single patient with different liposome preparations. The radiation hazard to the patient would be low.

The studies reported in this paper indicate that the distribution of $^{99m}$Tc-labelled liposomes in normal tissues of man and rats is similar. This included blood clearance, liver accumulation and excretion of the $^{99m}$Tc label (Richardson *et al.*, 1977). However, in man the similarity excludes tumour localization, except perhaps in the one case of Patient 1a with a primary hepatoma. It may be noted that this was *not* found to be reproducible (Study 1b) using a larger dose of liposomes. However, the second of these studies was made the day following the end of a course of cytotoxic chemotherapy and this possibly influenced the results.

In our rat study (Richardson *et al.*, 1977, 1978*b*) we reported that many factors influenced the tumour localization of the $^{99m}$Tc label. These included lipid composition, liposome charge, size and lipid fluidity. Further studies in cancer patients are required to examine the importance of each of these factors, as well as extending the range of tumours studied.

We wish to thank Professor K. D. Bagshawe for his helpful criticism and Mr A. Osifeso for preparing phosphatidylcholine. We acknowledge the Cancer Research Campaign for financial support.

### REFERENCES

ADAMS, D. H., JOYCE, G., RICHARDSON, V. J., RYMAN, B. E. & WISNIEWSKI, H. M. (1977) Liposome toxicity in the mouse central nervous system. *J. Neurol. Sci.*, **31**, 173.

ANGHILERI, L. J., FRUSIAN, N. & BRUCKSCH, K. P. (1976) *In vivo* distribution of $^{99m}$Tc labelled liposomes. *J. Nucl. Biol. Med.*, **20**, 165.

BANGHAM, A. D., STANDISH, M. M. & WATKINS, J. C. (1965) Diffusion of univalent ions across the lamellae of swollen phospholipids. *J. Mol. Biol.*, **13**, 238.

BELCHETZ, P. E., BRAIDMAN, I. P., CRAWLEY, J. C. W. & GREGORIADIS, G. (1977) Treatment of Gaucher's disease with liposome-entrapped glucocerebroside-β-glucosidase. *Lancet*, ii. 116.

BLACK, C. D. V. & GREGORIADIS, G. (1977) Interaction of liposomes with blood plasma proteins. *Biochem. Soc. Trans.*, **4**, 253.

BRUNI, A., TOFFANO, G., LEON, A. & BOCRATO, E. (1976) Pharmacological effects of phosphatidylserine liposomes. *Nature*, **260**, 331.

BUSCH, H., FUJIWARA, E. & FIRSZT, D. C. (1961) Studies on the metabolism of radioactive albumin in tumour bearing rats. *Cancer Res.*, **21**, 371.

COLLEY, C. M. & RYMAN, B. E. (1975) Liposomes as carriers *in vivo* for methotrexate. *Biochem. Soc. Trans.*, **3**, 157.

DAPERGOLAS, G., NEERUNJUN, E. D. & GREGORIADIS, G. (1976) Penetration of target areas in the rat by liposome-entrapped bleomycin glucose oxidase and inulin. *FEBS Lett.*, **63**, 235.

EASTY, G. C., YARNELL, M. M. & ANDREWS, R. D. (1964) The uptake of proteins by normal and tumour cells *in vitro*. *Br. J. Cancer*, **18**, 354.

GHOSE, T., NAIRN, R. C. & FOTHERGILL, J. E. (1962) Uptake of proteins by malignant cells. *Nature*, **196**, 1108.

GREGORIADIS, G. (1976) Carrier potential of liposomes in biology and medicine. *N. Engl. J. Med.*, **295**, 704.

GREGORIADIS, G. & RYMAN, B. E. (1972) Fate of protein-containing liposomes injected into rats. An approach to the treatment of storage diseases. *Eur. J. Biochem.*, **24**, 487.

GREGORIADIS, G., SWAIN, P., WILLS, E. J. & TAVILL, A. S. (1974) Drug-carrier potential of liposomes in cancer chemotherapy. *Lancet*, i, 1313.

JULIANO, R. L. & STAMP, D. (1978) Pharmacokinetics of liposome-encapsulated anti-tumour drugs. Studies with vinblastine, actinomycin D, cytosine arabinoside and daunomycin. *Biochem. Pharmacol.*, **27**, 21.

KOBAYASHI, T., KATAOKA, T., TSUKAGOSHI, S. & SAKURAI, Y. (1977) Enhancement of anti-tumour activity of 1-β-D-arabinofuranosylcytosine by encapsulation in liposomes. *Int. J. Cancer*, **20**, 581.

KOSLOSKI, M. J., ROSEN, F., MILHOLLAND, R. J. & PAPAHADJOPOULOS, D. (1978) Effect of lipid vesicle (liposome) encapsulation of methotrexate on its chemotherapeutic efficacy in solid rodent tumours. *Cancer Res.*, **38**, 2848.

LOKICH, J. J., DRUM, D. E. & KAPLAN, W. (1974) Hepatic toxicity of nitrosourea analogues. *Clin. Pharmacol. Ther.*, **16**, 363.

MAGAREY, C. J. & BAUM, M. (1970) Reticulo-endothelial activity in humans with cancer. *Br. J. Surg.*, **57**, 648.

MEGO, J. L. & MCQUEEN, J. D. (1965) The uptake of labelled proteins by particulate fractions of tumour and normal tissues after injection into mice. *Cancer Res.*, **25**, 865.

NEERUNJUN, E. D., HUNT, R. & GREGORIADIS, G. (1977) Fate of a liposome-associated agent injected into normal and tumour-bearing rodents: Attempts to improve localisation in tumour tissues. *Biochem. Soc. Trans.*, **5**, 1380.

PAPAHADJOPOULOS, D. (Ed.) (1978) Liposomes and their uses in biology and medicine. *Ann. N.Y. Acad. Sci.*, **308**, 1.

RICHARDSON, V. J., JEYASINGH, K., JEWKES, R. F., RYMAN, B. E. & TATTERSALL, M. H. N. (1977) Properties of [$^{99m}$Tc]technetium-labelled liposomes in normal and tumour-bearing rats. *Biochem. Soc. Trans.*, **5**, 290.

RICHARDSON, V. J., JEYASINGH, K., JEWKES, R. F., RYMAN, B. E. & TATTERSALL, M. H. N. (1978*a*) Possible tumour localisation of Tc-99m labelled liposomes: Effects of lipid composition, charge and liposome size. *J. Nucl. Med.*, **19**, 1049.

RICHARDSON, V. J., RYMAN, B. E., JEWKES, R. F., TATTERSALL, M. H. N. & NEWLANDS, E. S. (1978*b*) $^{99m}$Tc-labelled liposomes: Preparation of radiopharmaceutical and its distribution in a hepatoma patient. *Int. J. Nucl. Biol. & Med.*, **5**, 118.

RUTMAN, R. J., RITTER, C. A., AVADHANI, N. G. & HANSEL, J. (1977) Liposomal potentiation of the anti-tumour activity of 1-β-D-arabinofuranosylcytosine by encapsulation in liposomes. *Int. J. Cancer*, **20**, 581.

SEGAL, A. W., GREGORIADIS, G., LAVENDER, J. P., TARIN, D. & PETERS, T. J. (1976) Tissue and hepatic subcellular distribution of liposomes containing bleomycin after intravenous administration to patients with neoplasms. *Clin. Sci. Mol. Med.*, **51**, 421.

SHARBAUGH, R. J. & GROGAN, J. B. (1969) Suppression of reticulo-endothelial function in the rat with cyclophosphamide. *J. Bacteriol.*, **100**, 117.

STEGER, L. D. & DESNICK, R. J. (1977) Enzyme therapy VI: Comparative *in vivo* fates and effects on lysosomal integrity of enzyme entrapped in negatively and positively-charged liposomes. *Biochim. Biophys. Acta*, **464**, 530.

SUBRAMANIAN, G., RHODES, B. A., COOPER, J. F. & SODD, V. J. (Eds.) (1975) *Radiopharmaceuticals.* New York: The Society of Nuclear Medicine Inc.

TROUET, A. DE CAMPENEERE, D. D. & DE DUVE, C. (1972) Chemotherapy through lysosomes with a DNA-daunorubicin complex. *Nature* (*New Biol.*), **239**, 110.

TYRRELL, D. A., RYMAN, B. E., KEETON, B. R. & DUBOWITZ, V. (1976) Use of liposomes in treating Type II glycogenosis. *Br. Med. J.*, ii, 88.

TYRRELL, D. A., RICHARDSON, V. J. & RYMAN, B. E. (1977) The effect of serum protein fractions on liposome-cell interactions in cultured cells and the perfused rat liver. *Biochim. Biophys. Acta*, **497**, 469.